# Related factors and a predictive model for early neurological deterioration after mechanical thrombectomy for acute ischemic stroke due to large vessel occlusion

**DOI:** 10.3389/fneur.2026.1749495

**Published:** 2026-04-09

**Authors:** Yang Li, Yuanyuan Xue, Shuai Liu, Sifei Wang, Leilei Luo, Shuling Liu, Ming Wei

**Affiliations:** 1 Department of Neurosurgery, Tianjin Medical University, Tianjin, China; 2 Tianjin Key Laboratory of Cerebral Vascular and Neurodegenerative Diseases, Tianjin, China; 3 Department of Neurosurgery, Tianjin Huanhu Hospital, Tianjin, China

**Keywords:** early neurological deterioration, endovscular, large vessel occlusion, mechanical thrombectomy, stroke

## Abstract

**Background:**

Mechanical thrombectomy (MT) is an established reperfusion therapy for acute ischemic stroke due to large vessel occlusion (LVO-AIS) and has been proven to significantly improve 90-day functional outcomes. However, some patients still experience early neurological deterioration (END) despite successful recanalization. This study aimed to systematically identify independent risk factors for END after MT via retrospective cohort analysis and construct a nomogram by integrating laboratory and clinical characteristics.

**Methods:**

A total of 486 LVO-AIS patients with successful recanalization (eTICI≥2b) were first categorized as END (ΔNIHSS≥4) or non-END (ΔNIHSS<4) according to the change in the NIHSS score from baseline to 24 h post-procedure. The entire patient group was then randomly divided to obtain training (70%, *n* = 341) and validation (30%, *n* = 145) cohorts. A nomogram was constructed and subsequently validated.

**Results:**

We conducted a LASSO regression analysis on the clinical data of the patients in the modelling cohort and identified 6 disease characteristic variables. These variables were subsequently entered into a multivariate logistic regression model, which ultimately retained 5 independent predictors: smoking status (OR = 3.90, 95% CI 1.760–9.681, *p* = 0.002), platelet count (OR = 1.54, 95% CI 1.129–2.130, *p* = 0.007), systolic blood pressure (SBP) (OR = 1.80, 95% CI 1.300–2.543, *p* = 0.001), puncture-to-recanalization time (PRT) (OR = 1.57, 95% CI 1.084–2.346, *p* = 0.024), and the neutrophil-to-lymphocyte ratio (NLR) (OR = 1.86, 95% CI 1.311–2.703, *p* = 0.001). The prediction model showed good discriminative performance, with a C-index of 0.803 (95% CI: 0.738–0.868; *p* < 0.001). The area under the ROC curve (AUC) was 0.803 (95% CI 0.738–0.868) in the training cohort and 0.799 (95% CI 0.693–0.905; *p* < 0.001) in the validation cohort. The model also exhibited good calibration. The Hosmer–Lemeshow goodness-of-fit test showed no significant difference between the predicted and observed results (χ^2^ = 3.607, *p* = 0.891), and the mean absolute error of the calibration curve in the training cohort was 0.169.

**Conclusion:**

The constructed prediction model accurately estimated the risk of END in LVO-AIS patients who underwent MT with successful recanalization and may help optimize patient selection for endovascular therapy and provide reliable prognostic information.

## 1 Introduction

AIS is a major global cause of disability and mortality (1–3). A significant proportion of AIS cases result from LVO, which is associated with severe neurological deficits and poor outcomes because of the extensive brain territory at risk (4). MT has become the standard reperfusion therapy for LVO-AIS, substantially improving functional independence compared with medical management alone (5).

Despite high rates of successful recanalization, a subset of patients experience END within 24 h post-procedure. This devastating complication leads to a sharp increase in disability and mortality, highlighting the critical need for its early prediction and prevention (6, 7). The pathophysiology of END is multifactorial and involves processes such as reperfusion injury, microcirculatory failure, and intense inflammatory responses (8, 9). Several risk factors have been implicated, including clinical characteristics, imaging findings, and laboratory markers of inflammation (10–17). Although these associations exist, a robust, readily applicable tool that integrates key predictors to stratify END risk at the individual patient level is lacking. Therefore, the objective of this study was to develop and validate a practical predictive model for END following MT in LVO-AIS patients. Using a retrospective cohort design and machine learning techniques for variable selection, we constructed a nomogram to provide clinicians with a quantifiable means of identifying high-risk patients, thereby facilitating timely interventions and improving outcomes.

## 2 Methods

### 2.1 Study design and participants

This single-center, retrospective cohort study was conducted at a comprehensive stroke center in Tianjin, China. We screened consecutive patients with LVO-AIS who underwent MT between January 2023 and April 2024.

The inclusion criteria were (1) age ≥18 years; (2) a diagnosis of AIS according to the Chinese Guidelines for the Diagnosis and Treatment of Acute Ischemic Stroke (2018); (3) occlusion of the intracranial internal carotid artery, M1 or M2 segment of the middle cerebral artery, V4 segment of the vertebral artery, or basilar artery, confirmed by computed tomography angiography (CTA), magnetic resonance angiography (MRA), or digital subtraction angiography (DSA); and (4) successful recanalization after MT, defined as an expanded thrombolysis in cerebral infarction (eTICI) score of ≥2b.

**Figure 1:**
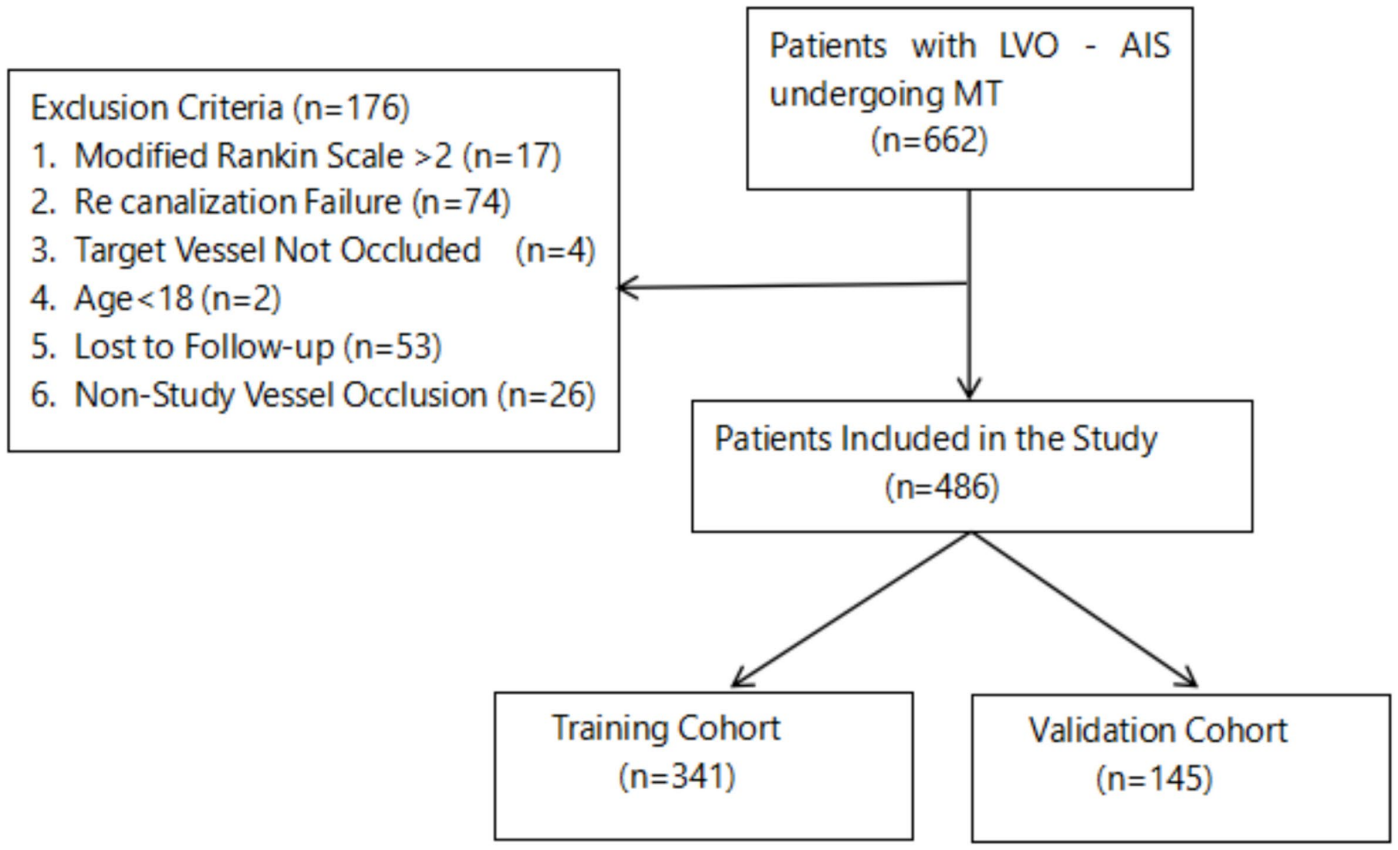
Study design and participants.

The exclusion criteria were (1) a prestroke modified Rankin scale (mRS) score >2; (2) failure of recanalization (eTICI<2b); (3) vascular stenosis without occlusion; (4) occlusion in nontargeted arteries (e.g., anterior cerebral artery or posterior cerebral artery); (5) incomplete key clinical or laboratory data; or (6) loss to follow-up (see Figure 1).

### 2.2 Data collection and variable definitions

Data were extracted from our prospectively maintained stroke registry and electronic medical records. The collected data included the following:

Demographics and medical history: age, sex, vascular risk factors (hypertension, diabetes, atrial fibrillation, or coronary heart disease), and history of smoking, alcohol consumption, transient ischemic attack (TIA), and stroke.

Baseline clinical and imaging characteristics: admission National Institutes of Health Stroke Scale (NIHSS) score and Alberta Stroke Program Early CT Score (ASPECTS).

Procedure details: puncture-to-recanalization time (PRT), onset-to-puncture time (OPT), onset-to-recanalization time (ORT), number of retrieval attempts, MT technique (aspiration alone vs. stent retriever ± aspiration), and degree of residual stenosis.

Laboratory parameters: baseline systolic and diastolic blood pressure (SBP and DBP), blood glucose, platelet count, white blood cell count (WBC), and differential counts for calculating the neutrophil-to-lymphocyte ratio (NLR), platelet-to-lymphocyte ratio (PLR), lymphocyte-to-monocyte ratio (LMR), systemic inflammation response index (SIRI), and systemic immune-inflammation index (SII). Coagulation parameters and lipid profiles were also collected.

The primary outcome was END, defined as an increase of ≥4 points in the NIHSS score from baseline to within 24 h after MT.

### 2.3 Statistical analysis

Statistical analyses were performed using R software (version 4.4.2) and IBM SPSS Statistics (version 27.0). Continuous variables are presented as the mean ± standard deviation (SD) if normally distributed or as the median with interquartile range (IQR) if nonnormally distributed and were compared using Student’s *t*-test or the Mann–Whitney U test, respectively. Categorical variables are summarized as frequencies (percentages) and were compared using the chi-square test or Fisher’s exact test.

The total cohort was randomly split into a training cohort (70%) and a validation cohort (30%) using a computer-generated random sequence. In the training cohort, least absolute shrinkage and selection operator (LASSO) regression with 10-fold cross-validation was employed for variable selection to prevent overfitting. The optimal tuning parameter (*λ*) was selected via the minimum criterion (λ.min) and the one-standard-error rule (λ.1se); the λ.1se value was chosen to obtain a more parsimonious model. Multicollinearity among the LASSO-selected variables was assessed using the variance inflation factor (VIF), with a VIF < 10 considered acceptable. The linearity of continuous variables with the logit of the outcome was assessed using restricted cubic splines (RCS). For variables meeting the linearity assumption (Pnon-linear > 0.05), they were included in the multivariable model in their original continuous form to preserve predictive information.

Variables selected by LASSO regression were subsequently entered into a multivariable logistic regression model to establish the final prediction model for END and to calculate odds ratios (ORs) with 95% confidence intervals (CIs). A nomogram based on this final model was constructed using the rms package in R.

The discriminative ability of the model was evaluated by the area under the receiver operating characteristic curve (AUC) and the concordance index (C-index). Calibration was assessed using calibration plots and the Hosmer–Lemeshow goodness-of-fit test. The performance of the model was rigorously validated in the independent validation cohort. A two-tailed *p* < 0.05 was considered to indicate statistical significance.

### 2.4 Ethical considerations

Data underpinning the findings of this study can be obtained from the corresponding author upon reasonable request. This study was approved by the Institutional Ethics Committee, and all the subjects provided written informed consent.

## 3 Results

### 3.1 Patient characteristics

Between January 2023 and April 2024, 662 LVO-AIS patients who underwent MT were initially enrolled. After applying the exclusion criteria—including revascularization failure (eTICI<2b; *n* = 74), prestroke mRS > 2 (*n* = 17), loss to follow-up (*n* = 53), age<18 years (*n* = 2), vascular stenosis without occlusion (*n* = 4), and occlusion in nontarget arteries [anterior cerebral artery (ACA), posterior cerebral artery (PCA), or posterior inferior cerebellar artery (PICA); *n* = 26]—a final cohort of 486 patients was included in the analysis. The baseline characteristics were well balanced between the two cohorts (all *p* > 0.05) (Table 1), indicating successful randomization.

**Table 1:** Baseline characteristics of LVO-AIS patients in the training and validation cohorts.

Indicators	Total cases (*n* = 486)	Training cohort (*n* = 341)	Validation cohort (*n* = 145)	*P*-value
END, n(%) demographic characteristics	75(15.4)	53(15.5)	22(15.2)	0.918
Age, median (IQR)	65(57.71)	65(56.71)	66(57.5)	0.991
Sex, n(%)				0.821
Female	124(25.5)	88(25.8)	36(24.8)	
Male	362(74.5)	253(74.2)	109(75.2)	
Vascular risk factors, n (%)				
Hypertension	317(65.2)	220(64.5)	97(66.9)	0.614
Diabetes	138(28.4)	99(29.0)	39(26.9)	0.633
Atrial fibrillation	205(42.2)	140(41. 1)	65(44.8)	0.441
Coronary disease	71(14.6)	49(14.4)	22(15.2)	0.819
History of TIA or stroke	78(16.0)	55(16. 1)	23(15.9)	0.942
History of smoking	141(29.0)	104(30.5)	37(25.5)	0.268
History of alcohol consumption	294(60.5)	213(62.5)	81(55.9)	0.173
Admission NIHSS, median (IQR)	275(56.6)	202(59.2)	73(50.3)	0.070
NIHSS at 24 h post-MT, median (IQR)	12(7.16)	11(6.16)	12(8.16)	0.586
ASPECTs at admission, median (IQR)	10(7.16)	10(7.16)	10(6.5.16)	0.790
TOAST classification, n(%)				0.968
LAA	402(82.7)	283(83.0)	119(82. 1)	
CE	65(13.4)	45(13.2)	20(13.8)	
Other types	19(3.9)	13(3.8)	6(4. 1)	
Occluded vessel, n(%)				0.425
ICA	69(14.2)	42(12.3)	27(18.6)	
MCA	249(51.2)	179(52.5)	70(48.3)	
ICA + MCA tandem lesions	66(13.6)	45(13.2)	21(14.5)	
VA-V4	17(3.5)	11(3.2)	6(4. 1)	
BA	44(9. 1)	32(9.4)	12(8.3)	
VA + BA tandem lesions	41(8.4)	32(9.4)	9(6.2)	
Antithrombotic therapy, n (%)	22(4.5)	14(4. 1)	8(5.5)	0.493
Antiplatelet therapy, n(%)	97(20.0)	69(20.2)	28(19.3)	0.816
Anticoagulant therapy, n(%)	13(2.7)	6(1.8)	7(4.8)	0.055
OPT, median (IQR)	284(191.75, 402.00)	283(187.00, 405)	285(198.5, 388)	0.962
ORT, median (IQR)	598(413.75, 891.75)	627(431, 916)	579(400, 827.5)	0.100
PRT, median (IQR)	130(100, 180)	131(100, 180)	130(96.5, 177)	0.827
Technique, n(%)				0.895
Aspiration	99(20.4)	70(20.5)	29(20.0)	
Stent ± aspiration	387(79.6)	271(79.5)	116(80.0)	
Number of passes, median (I QR)	2(1.3)	2(1.3)	1(1.3)	0.518
Degree of residual stenosis, n (%)				0.978
None	203(41.8)	144(42.2)	59(40.7)	
Mild	151(31. 1)	105(30.8)	46(31.7)	
Moderate	100(20.6)	69(20.2)	31(21.4)	
Severe	32(6.6)	23(6.7)	9(6.2)	
Laboratory parameters				
SBP, mmHg, median (IQR)	145(128.75, 162)	146(131, 162.5)	140(125, 158)	0.052
DBP, mmHg, median (IQR)	85(75, 94.25)	85(75.94)	86(75.5, 95.5)	0.565
Blood glucose, median (IQR)	7.89(6.6875, 9.8)	7.9(6.69, 9.92)	7.83(6.655, 9.51)	0.694
Platelet count, median (IQR)	211(179.75, 241)	212(180, 242)	207(179, 238.5)	0.793
WBC, median (IQR)	9. 155(7.07, 11.3325)	9.3(7.065, 11.32)	8.9(7.10, 11.36)	0.883
NLR, median (IQR)	5.71(3.50, 9.15)	5.63(3.45, 9.08)	5.95(3.61, 9.30)	0.574
PLR, median (IQR)	161.45(118.9, 0, 229.99)	159.75(120.5 7, 226.69)	168.85(114.9 5, 233.71)	0.633
LMR, median (IQR)	3.255(2.25, 4.59)	3.33(2.20, 4.65)	3. 16(2.35, 4.33)	0.674
SIRI, median (IQR)	2. 19(1.20, 3.79)	2. 19(1.18, 3.80)	2.28(1.32, 3.86)	0.746
SII, median (IQR)	1191.99(692. 52, 1871. 13)	1192.71(664. 65, 1853.67)	1174.93(735. 70, 1958.03)	0.671
D dimer, median (IQR)	1.41(0.72, 3.62)	1.33(0.73, 3.9)	1.6(0.67, 3.51)	0.783
PT, median (IQR)	11. 10(10.60, 11.60)	11. 10(10.60, 11.60)	11. 10(10.60, 11.65)	0.817
APTT, median (IQR)	23.4(20.88, 25.63)	23.40(20.90, 25.55)	22.90(20.80, 25.90)	0.611
INR, median (IQR)	0.94(0.90, 0.99)	0.94(0.90, 0.99)	0.94(0.90, 0.99)	0.817
TC, median (IQR)	4.68(3.98, 55.35)	4.67(3.94, 5.34)	4.72(4.11, 5.38)	0.595
TG, median (IQR)	1. 1(0.82, 1.52)	1. 1(0.84, 1.51)	1. 13(0.78, 1.53)	0.896
LDL, mean (SD)	3.00(0.80)	3.00(0.80)	3.00(0.80)	0.918
HDL, median (IQR)	1. 14(0.99, 1.31)	1. 14(0.99, 1.30)	1. 11(0.96, 1.35)	0.831
90 days mRS, n(%)				0.624
≤2	263(54. 1)	187(54.8)	76(52.4)	
>2	223(45.9)	154(45.2)	69(47.6)	
Death, n(%)	29(6.0)	22(6.5)	7(4.8)	0.489

END, Early neurological deterioration; TIA, Transient ischemic attack; NIHSS, National Institutes of Health stroke scale; ASPECTS, Alberta stroke program early CT score; TOAST, Trial of Org 10,127 in acute stroke treatment; LAA, Large-artery atherosclerosis; CE, Cardio-embolism; ICA, Internal Carotid Artery; MCA, Middle Cerebral Artery; VA, Vertebral Artery; BA, Basilar Artery; OPT, Onset to puncture; ORT, Onset to recanalization; PRT, Puncture to recanalization; DBP, Diastolic blood pressure; WBC, White Blood Cell; NLR, Neutrophil to lymphocyte ratio; PLR, Platelet to lymphocyte ratio; LMR, Lymphocyte to monocyte ratio; SIRI, Systemic inflammation response index; SII, Systemic immune-inflammation index; PT, Prothrombin time; APTT, Activated partial thromboplastin time; INR, International normalized ratio; TC, Total Cholesterol; TG, Triglyceride; LDL, Low-Density Lipoprotein.

On the basis of the change in the NIHSS score from admission to 24 h post-treatment, the participants were categorized into two groups: the END group (ΔNIHSS ≥ 4) and the non-END group (ΔNIHSS<4). The non-END group included 288 patients (84.5%), including 72 females (25.0%) and 216 males (75.0%). In this group, 169 patients (58.7%) had a smoking history. The median NIHSS score at 24 h post-MT was 10 (IQR: 6–14), the median puncture-to-recanalization time (PRT) was 128.5 min (IQR: 100–177.5), and the mean baseline systolic blood pressure (SBP) was 146.2 ± 22.05 mmHg. The END group included 53 patients (15.5%), including 16 females (30.2%) and 37 males (69.8%). Among them, 44 patients (83.0%) had a smoking history. The median NIHSS score at 24 h post-MT was 17 (IQR: 12–25), the median PRT was 144 min (IQR: 120–188), and the mean baseline SBP was 156.79 ± 24.54 mmHg. Comparisons between the END and non-END groups revealed significant differences in several baseline characteristics: the END group had a significantly greater proportion of smokers (83.0% vs. 58.7%, *p* < 0.001), higher 24-h post-MT NIHSS scores [median 17 (IQR 12–25) vs. 10 (6–14), *p* < 0.001], longer baseline puncture-to-recanalization times [PRT; median 144 (IQR 120–188) vs. 128.5 (100–177.5) minutes, *p* = 0.008], and higher baseline SBPs (156.79 ± 24.54 vs. 146.2 ± 22.05 mmHg, *p* = 0.002). Significant differences were also observed in the baseline laboratory parameters (Table 2). Compared with the non-END group, the END group had a higher mean baseline platelet count (231.40 ± 61.15 vs. 209.83 ± 43.01 × 10^9^/L, *p* = 0.017), median baseline NLR [8.42 (IQR 5.45–12.17) vs. 5.12 (2.92–8.57), *p* < 0.001], PLR [192.21 (139.99–284.77) vs. 153.88 (116.87–219.16), *p* = 0.005], SIRI [3.36 (2.07–6.19) vs. 2.01 (1.06–3.42), *p* < 0.001], and SII [1853.89 (1096.32–2803.19) vs. 1092.05 (625.39–1730.01), *p* < 0.001]. Conversely, the LMR was lower in the END group [2.49 (1.87–3.79) vs. 3.43 (2.31–4.81), *p* = 0.002].

**Table 2:** Comparison of baseline characteristics between non-END and END groups in the training cohort.

Clinical baseline characteristics	Total cases (*n* = 341)	Non-END group (*n* = 288)	END group (*n* = 53)	*P*-value
Age, median (IQR)	65(56.71)	65(55.25, 71)	65(58, 70.5)	0.411
Sex, n(%)				0.428
Female	88(25.8)	72(25.0)	16(30.2)	
Male	253(74.2)	216(75.0)	37(69.8)	
Hypertension	220(64.5)	182(63.2)	38(71.7)	0.234
Diabetes mellitus	99(29.0)	86(29.9)	13(24.5)	0.432
Hyperkinemia	140(41. 1)	115(39.9)	25(47.2)	0.325
Atrial fibrillation	49(14.4)	41(14.2)	8(15. 1)	0.870
Coronary disease	55(16. 1)	48(16.7)	7(13.2)	0.529
History of TIA or stroke	104(30.5)	84(29.2)	20(37.7)	0.213
History of smoking	213(62.5)	169(58.7)	44(83.0)	<0.001
History of alcohol consumption	202(62.5)	169(58.7)	33(62.3)	0.626
Admission NIHSS, median (IQR)	11(7, 16)	12(7, 16)	9(6.5, 14)	0.165
NIHSS within 24 h after MT, median (IQR)	10(7, 16)	10(6, 14)	17(12.25)	<0.001
ASPECTs at admission, median (IQR)	7(5.8)	7(5.8)	6(5.7)	0.586
TOAST classification, n(%)				0.907
LAA	283(83.0)	238(82.6)	45(84.9)	
CE	45(13.2)	39(13.5)	6(11.3)	
Other types	13(3.8)	11(3.8)	2(3.8)	
Bleeding artery, n(%)				0.256
ICA	42(12.3)	37(12.8)	5(9.4)	
MCA	179(52.5)	156(54.2)	23(43.4)	
ICA + MCA tandem lesions	45(13.2)	36(12.5)	9(17.0)	
VA-V4	11(3.2)	10(3.5)	1(1.9)	
BA	32(9.4)	26(9.0)	6(11.3)	
VA + BAtandem lesions	32(9.4)	23(8.0)	9(17.0)	
Intravenous thrombolysis, n(%)	14(4. 1)	12(4.2)	2(3.8)	0.895
Antiplatelet therapy, n(%)	69(20.2)	58(20. 1)	11(20.8)	0.918
Anticoagulation therapy, n(%)	6(1.8)	5(1.7)	1(1.9)	0.939
OPT, median (IQR)	283(187, 405)	282(193.5, 405)	295(163.5, 446.5)	0.512
ORT, median (IQR)	627(431, 916)	630(433, 935)	596(420, 866)	0.520
PRT, median (IQR)	131(100, 180)	128.5(100, 17 7.5)	144(120, 188)	0.008
MT technique, n(%)				0.248
Aspiration	70(20.5)	56(19.4)	14(26.4)	
Stent ± aspiration	271(79.5)	232(80.6)	39(73.6)	
Number of take-stops, median (I QR)	2(1.3)	2(1.3)	2(1.3)	0.190
Residual stricture severity, n(%)				0.944
None	144(42.2)	120(41.7)	24(45.3)	
Mild	105(30.8)	90(31.3)	15(28.3)	
Moderate	69(20.2)	59(20.5)	10(18.9)	
Severe	23(6.7)	19(6.6)	4(7.5)	
Laboratory examination				
SBP, mean (SD)	147.85(22.74)	146.20(22.05)	156.79(24.54)	0.002
DBP, mean (SD)	84.56(13. 13)	84.73(13.44)	83.62(11.33)	0.572
Blood glucose, median (IQR)	7.90(6.69, 9.92)	7.90(6.64, 9.82)	8.40(6.82, 11.46)	0.291
Platelet count, mean, mean (SD)	213. 18(46.84)	209.83(43.01)	231.40(61. 15)	0.017
NLR, median (IQR)	5.63(3.45, 9.08)	5. 12(2.92, 8.57)	8.42(5.45, 12.17)	<0.001
PLR, median (IQR)	159.75(120.5 7, 226.69)	153.88(116.87, 219.16)	192.21(139.99, 284.77)	0.005
WBC, median (IQR)	9.30(7.07, 11.32)	9.07(7.00, 11.24)	9.93(7.71, 12.38)	0.198
LMR, median (IQR)	3.33(2.20, 4.65)	3.43(2.31, 4.8 1)	2.49(1.87, 3.79)	0.002
SIRI, median (IQR)	2. 19(1.18, 3.80)	2.01(1.06, 3.42)	3.36(2.07, 6.19)	<0.001
SII, median (IQR)	1192.71(664.6 5, 1853.67)	1092.05(625. 39, 1730.01)	1853.89(10960.32, 2803.19)	<0.001
D dimer, median (IQR)	1.33(0.73, 3.69)	1.28(0.68, 3.37)	1.63(0.87, 4.32)	0.124
PT, median (IQR)	11. 10(10.60, 1 1.60)	11. 10(10.60, 1 1.58)	11.00(10.50, 1 1.60)	0.377
APTT, median (IQR)	23.40(20.90, 2 5.55)	23.50(20.93, 25.50)	23. 10(20.80, 2 6.25)	0.833
INR, median (IQR)	0.94(0.90, 0.99)	0.94(0.90, 0.99)	0.94(0.89, 0.99)	0.429
TC, mean (SD)	4.73(1.08)	4.74(1. 10)	4.65(1.02)	0.602
TG, median (IQR)	1. 1(0.84, 1.51)	1. 10(0.86, 1.55)	1. 10(0.81, 1.47)	0.749
LDL, median (IQR)	2.96(2.43, 3.47)	2.97(2.4125, 3.47)	2.89(2.49, 3.26)	0.789
HDL, median (IQR)	1. 14(0.99, 1.30)	1. 14(0.99, 1.29)	1. 15(0.98, 1.33)	0.867
eTICI, n (%)				0.687
2b	64 (18.8)	53 (18.4)	11 (20.8)	
2c–3	277 (81.2)	235 (81.6)	42 (79.2)	

END, Early neurological deterioration; TIA, Transient ischemic attack; NIHSS, National Institutes of Health stroke scale; ASPECTS, Alberta stroke program early CT score; TOAST, Trial of Org 10,127 in acute stroke treatment; LAA, Large-artery atherosclerosis; CE, Cardio-embolism; ICA, Internal Carotid Artery; MCA, Middle Cerebral Artery; VA, Vertebral Artery; BA, Basilar Artery; OPT, Onset to puncture; ORT, Onset to recanalization; PRT, Puncture to recanalization; DBP, Diastolic blood pressure; WBC, White Blood Cell; NLR, Neutrophil to lymphocyte ratio; PLR, Platelet to lymphocyte ratio; LMR, Lymphocyte to monocyte ratio; SIRI, Systemic inflammation response index; SII, Systemic immune-inflammation index; PT, Prothrombin time; APTT, Activated partial thromboplastin time; INR, International normalized ratio; TC, Total Cholesterol; TG, Triglyceride; LDL, Low-Density Lipoprotein; eTICI, expanded Treatment In Cerebral Ischemia. Continuous variables are expressed as mean ± SD or median [IQR]. Comparison between groups was performed using the Independent Samples t-test or the Mann–Whitney U test, as appropriate.

### 3.2 Construction of the END prediction model

**Figure 2:**
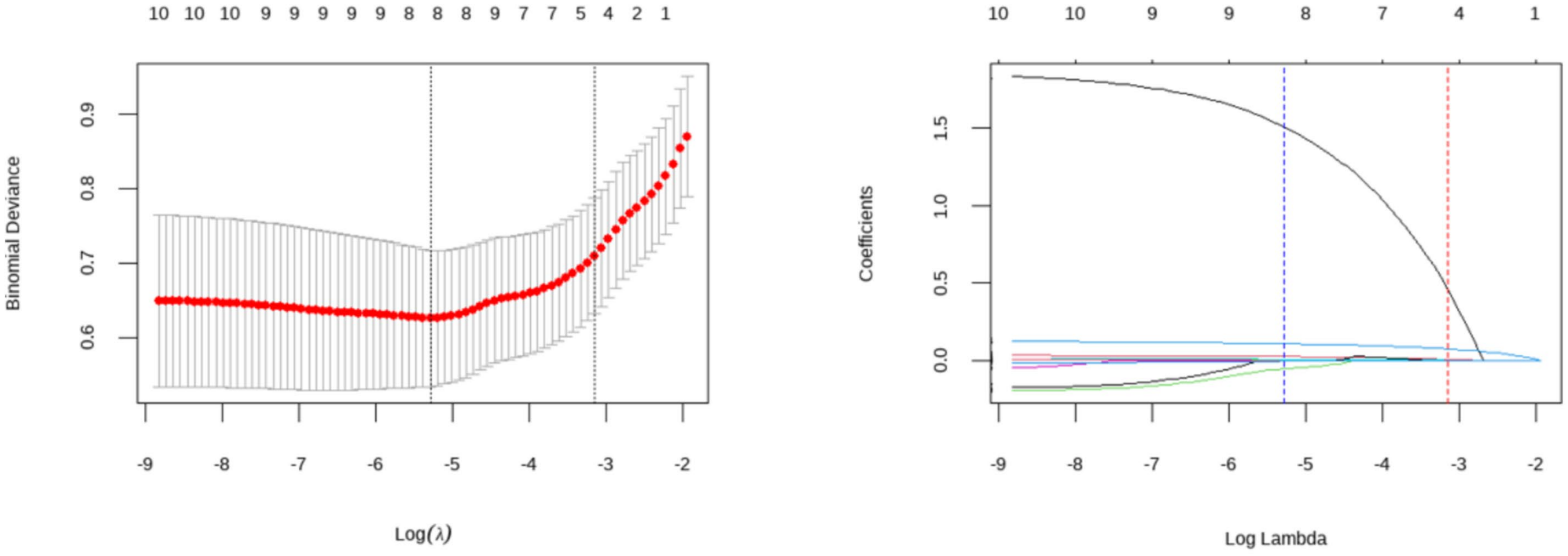
Feature variable selection using LASSO regression.

We first performed univariate logistic regression analysis on the 341 patients in the training cohort to identify potential predictor variables. To improve model stability, reduce redundancy, and prevent overfitting, we used LASSO regression for feature selection. Tenfold cross-validation was used to determine the optimal regularization parameter (*λ*) for the LASSO model. The values λ.min = 0.005 and λ.1se = 0.043 were identified (see Figure 2). To construct a parsimonious model, we selected λ.1se = 0.043 as the threshold for variable screening. Using this approach, six predictors were identified: smoking status, platelet count, systolic blood pressure (SBP), puncture-to-recanalization time (PRT), the neutrophil-to-lymphocyte ratio (NLR), and the lymphocyte-to-monocyte ratio (LMR).

To assess multicollinearity among the variables selected by LASSO regression, the variance inflation factor (VIF) was calculated for each variable. All VIF values were below 10, indicating that severe multicollinearity was not present among the predictors. Therefore, all six variables were included in the subsequent multivariate logistic regression analysis (Table 3).

**Table 3:** Assessment of multicollinearity.

Variable	VIF
Smoke	1.032
Platelet count	1.045
SBP	1.074
PRT	1.014
NLR	1.286
LMR	1.275

SBP, Systolic blood pressure; PRT, Puncture to recanalization; NLR, Neutrophil to lymphocyte ratio; LMR, Lymphocyte to monocyte ratio.

**Figure 3:**
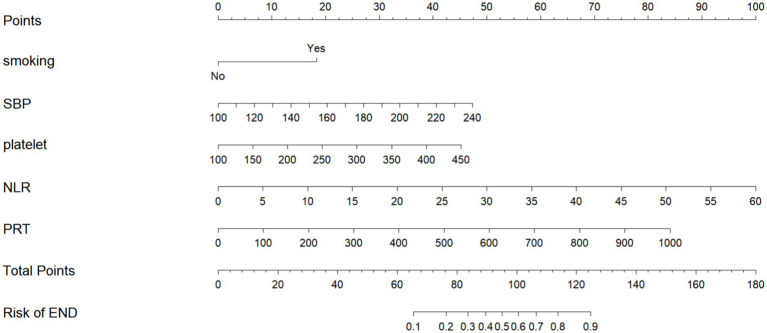
Nomogram for predicting the risk of early neurological deterioration (END) in patients with LVO-AIS.

The multivariate logistic regression model included the six variables selected by LASSO regression: smoking status, baseline platelet count, baseline SBP, baseline PRT, baseline NLR, and baseline LMR. The results demonstrated that smoking (OR = 3.90, 95% CI 1.76–9.68, *p* = 0.002), platelet count (per × 10^9^/L; OR = 1.54, 95% CI 1.13–2.13, *p* = 0.007), SBP (per mmHg; OR = 1.80, 95% CI 1.30–2.54, *p* < 0.001), PRT (per minute; OR = 1.57, 95% CI 1.08–2.35, *p* = 0.024), and NLR (OR = 1.86, 95% CI 1.31–2.70, *p* < 0.001) were independent predictors of END after MT. In contrast, the lymphocyte-to-monocyte ratio (LMR) was not significantly associated with END (OR = 0.81, 95% CI 0.465–1.227, *p* = 0.387) (Table 4). The independent predictors were used to construct a nomogram to predict END (Figure 3). This nomogram predicts the risk of END within 24 h after MT in LVO-AIS patients to use it, points are given for each variable value on the corresponding axis. The sum of these points yields a total score, which corresponds to a predicted probability of END on the bottom axis. For example, a patient with a smoking history, platelet count of 200 × 10^9^/L, SBP of 160 mmHg, PRT of 100 min, and NLR of 5 would have an estimated END risk of approximately 32%.

**Table 4:** Multivariate logistic regression analysis of END model after MT.

Predictor	OR	95%CI	*P*-value
Smoke	3.90	1.760–9.681	0.002
Platelet count	1.54	1.128–2.130	0.007
SBP	1.80	1.300–2.543	0.001
PRT	1.57	1.084–2.346	0.024
NLR	1.86	1.311–2.703	0.001
LMR	0.81	0.465–1.227	0.387

SBP, Systolic blood pressure; PRT, Puncture to recanalization; NLR, Neutrophil to lymphocyte ratio; LMR, Lymphocyte to monocyte ratio.

### 3.3 Evaluation and validation of the END clinical prediction model

**Figure 4:**
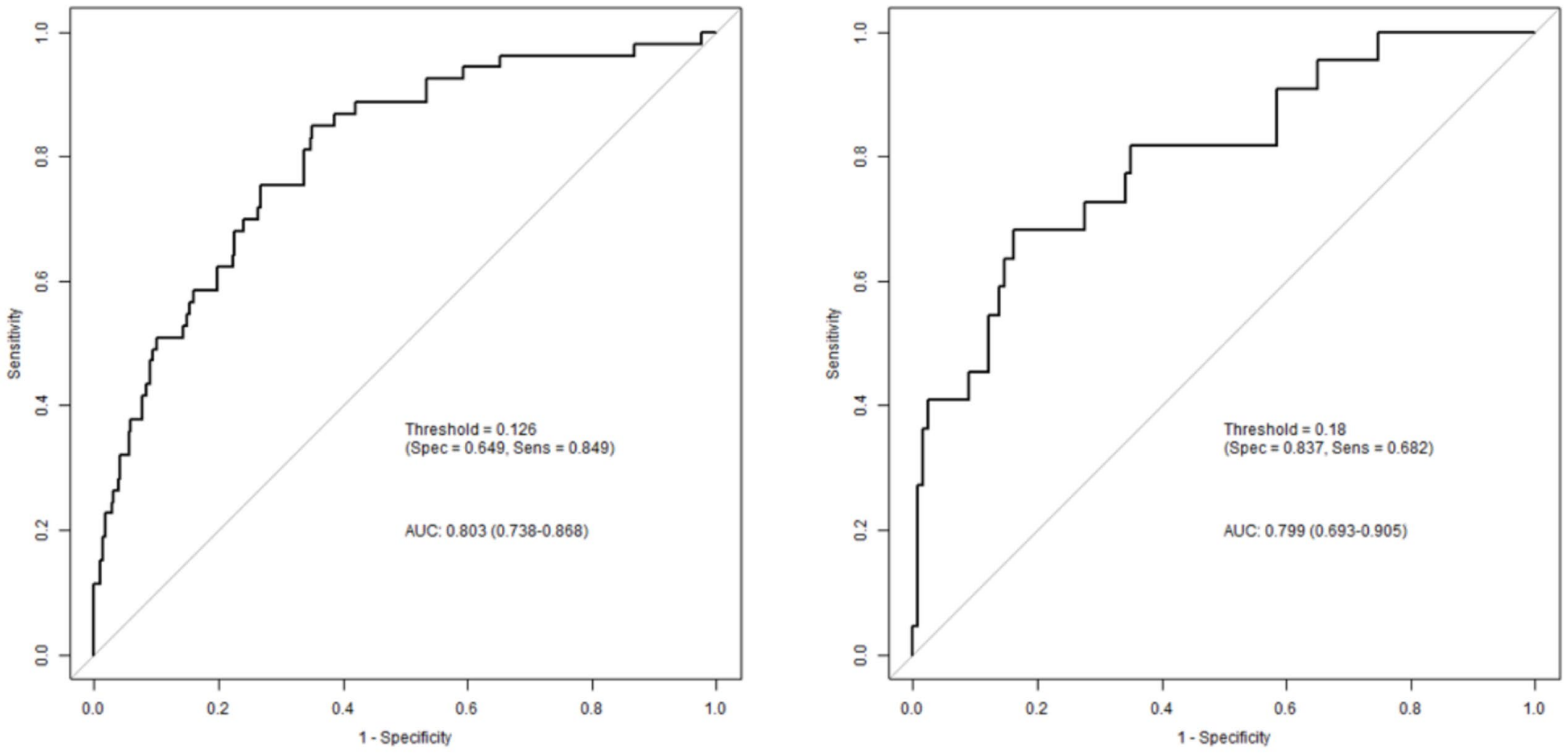
Evaluation of the discrimination of the prediction model using receiver operating characteristic curve (ROC curve).

The discriminative ability of the model, defined as its ability to distinguish between patients who will and will not develop END, was evaluated using several metrics. Discrimination was assessed using the C-index, area under the receiver operating characteristic curve (AUC), sensitivity, and specificity. Higher values indicate better discriminative performance. The model exhibited good discrimination. The C-index (equivalent to the AUC for binary outcomes) was 0.803 (95% CI 0.738–0.868; *p* < 0.001) in the training cohort, with a sensitivity of 0.849 and a specificity of 0.649. In the validation cohort, the AUC was 0.799 (95% CI 0.693–0.905; *p* < 0.001), with a sensitivity of 0.682 and a specificity of 0.837. The difference in the AUC between the training and validation cohorts was not statistically significant (*p* = 0.951), indicating consistent discriminative performance (Figure 4).

**Figure 5:**
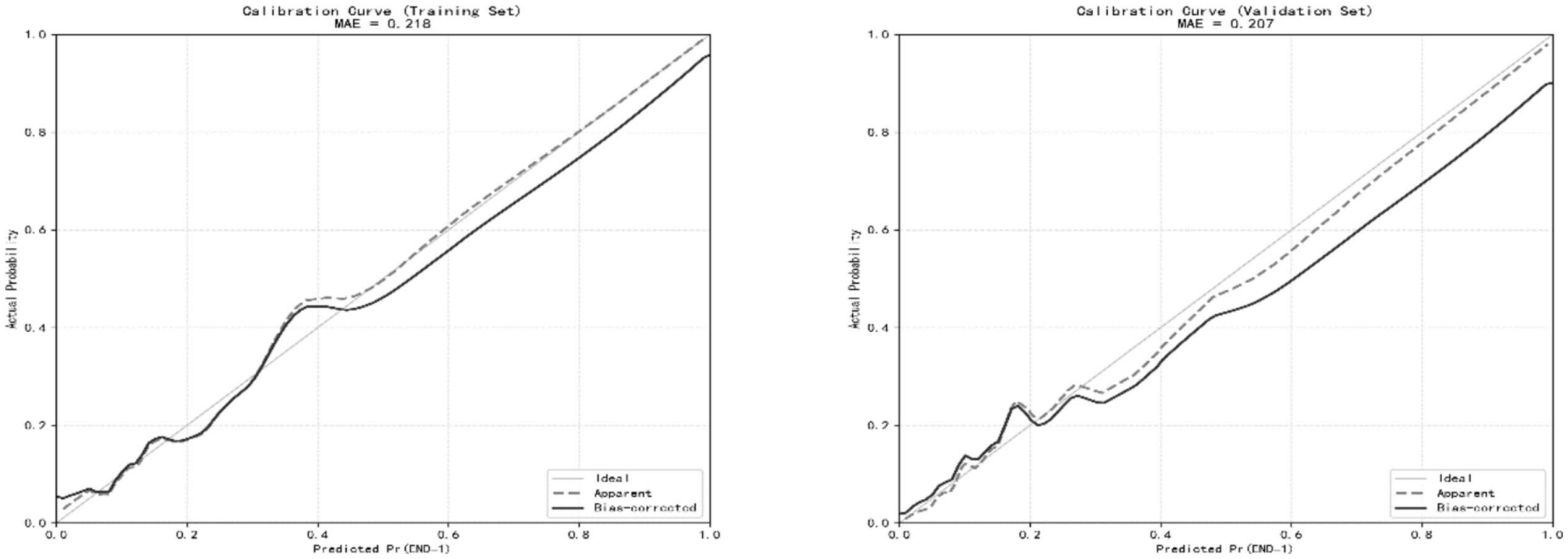
Calibration curves of the END prediction model in the training and validation cohorts.

The Hosmer-Lemeshow goodness-of-fit test was nonsignificant (χ^2^ = 3.607, *p* = 0.891), indicating that there was no significant deviation between the predicted and observed risks. The calibration curves for both cohorts closely approximated the 45-degree line of perfect agreement (Figure 5). The mean absolute error was 0.218 in the training cohort and 0.207 in the validation cohort, indicating good calibration performance.

## 4 Discussion

The principal finding of our study is the development and internal validation of a nomogram that accurately predicts the risk of END within 24 h after MT in patients with LVO-AIS. This practical model incorporates five readily obtainable clinical and procedural variables: smoking history, SBP, increased platelet count, PRT, and the NLR.

The inclusion of these specific variables is strongly supported by their interconnected pathophysiological roles in cerebral ischemia–reperfusion injury, a core mechanism of END. Our findings reinforce that END is not a monolithic entity but rather a multifactorial syndrome.

The strong independent association of a higher NLR with END underscores the central role of neuroinflammation. Neutrophils, which are rapidly recruited to the ischemic penumbra, contribute to microvascular occlusion, and reactive oxygen species and enzymes such as MMP-9 disrupt the blood–brain barrier, leading to edema and hemorrhagic transformation (18–20). Concurrent lymphocytopenia, reflecting stress-induced immunosuppression, compromises host defense and repair mechanisms, creating a deleterious cycle that exacerbates neuronal injury (21–24). The link between elevated baseline SBP and END can be explained by impaired cerebral autoregulation. Chronic hypertension damages the endothelium, and in the acute phase, this translates to an inability to control cerebral perfusion. This can exacerbate edema in reperfused territories and impair collateral flow, facilitating the expansion of the infarct core. Prolonged PRT is a direct metric of delayed reperfusion. Our results align with the established concept that “time is brain,” confirming that every minute of delay extends the duration of ischemia, accelerating the irreversible conversion of the penumbra into infarcted tissue and triggering apoptotic pathways (25–27). A smoking history and an increased platelet count collectively point to a pro-thrombotic and proinflammatory milieu. Smoking synergistically damages the endothelium, promotes hypercoagulability, and reduces oxygen-carrying capacity, while activated platelets not only contribute to thrombus formation but also release inflammatory mediators that amplify the ischemic cascade (28–30). The independent association of an elevated platelet count with END suggests mechanisms extending beyond a simple pro-thrombotic state. Beyond mediating thrombus formation, activated platelets act as key inflammatory coordinators that release proinflammatory cytokines and express surface adhesion molecules like P-selectin. These processes facilitate platelet-leukocyte aggregation, leading to microvascular plugging and the no-reflow phenomenon. Such microcirculatory failure, coupled with platelet-induced blood–brain barrier disruption, accelerates tissue injury even after successful macrovascular recanalization (28, 31).

Our model corroborates and integrates several risk factors previously reported in isolation. The significance of the NLR (32) and SBP (33) aligns well with the literature, emphasizing the effects of inflammation and hemodynamics on stroke outcomes. Similarly, the importance of PRT is a consistent theme across thrombectomy studies (34–36). Our model demonstrated robust discriminative power (AUC: 0.803), outperforming existing tools for unexplained END after EVT (AUC: 0.724; Girot et al. (37)) and NLR-based models for anterior circulation stroke (AUC: 0.72; Zhao et al. (38)). This incremental value likely stems from our focus on the successfully recanalized cohort and the integration of PRT and NLR, which collectively capture the dynamic interplay between procedural efficiency and post-ischemic inflammation better than baseline-only parameters. However, our multivariate model did not identify other previously reported factors, such as advanced age, hyperglycemia, or stroke etiology, as independent predictors. The exclusion of baseline risks such as age and glucose may stem from our specific cohort of successfully recanalized patients (eTICI ≥2b). In this context, acute procedural factors (PRT) and post-ischemic inflammation (NLR) may exert a more dominant influence on END, potentially masking traditional demographic or metabolic effects. Furthermore, the strong predictive power of the five selected variables might have superseded these factors during the parsimonious modeling process (9, 27). The primary clinical translation of our study lies in the developed nomogram. Converting a complex statistical model into a user-friendly visual tool enables rapid, individualized risk stratification at the bedside. Decision Curve Analysis (DCA) confirmed the clinical utility of our nomogram, showing a superior net benefit within a threshold range of 10–60% compared to alternative strategies. We identified a 20% risk threshold as a pragmatic trigger for clinical intervention. Patients above this cutoff should receive intensified Neuro-ICU monitoring (e.g., hourly NIHSS checks) and optimized hemodynamic management. This moves beyond mere prediction towards actionable clinical management. Identifying high-risk patients upon successful recanalization allows for intensified monitoring in specialized settings and prompt investigation of the causes of neurological decline and provides a rationale for future trials testing preemptive therapies, such as targeted anti-inflammatory regimens or stringent blood pressure control, in this vulnerable subgroup.

Our study has several limitations. First, the retrospective and single-center design may introduce selection bias and limit the generalizability of our findings. Although our internal validation demonstrated robust and consistent performance, the retrospective, single-center design may limit the generalizability of the nomogram. Therefore, large-scale, prospective, multi-center studies are mandatory to externally validate the reliability and clinical utility. Second, although we included a comprehensive set of variables, the performance of the model might be further refined by incorporating more advanced imaging biomarkers or dynamic laboratory trends. Nevertheless, a key strength of our model is its reliance on routinely available parameters, enhancing its immediate potential for real-world applications.

## 5 Conclusion

In this cohort of LVO-AIS patients with successful post-MT reperfusion, we identified five independent predictors of early neurological deterioration (END): elevated baseline systolic blood pressure, increased platelet count, smoking history, prolonged PRT, and an increased NLR. A clinically applicable nomogram incorporating these variables demonstrated robust predictive performance, accurately stratifying the risk of END within 24 h after the procedure. These findings enable early identification of high-risk individuals and provide an evidence-based framework for implementing targeted preventive measures and personalized management strategies.

## Data Availability

The original contributions presented in the study are included in the article/supplementary material, further inquiries can be directed to the corresponding authors.
